# The effect of low Al concentration on the electronic structure and thermoelectric properties of Al_x_Ga_1−x_N/GaN heterojunctions

**DOI:** 10.1038/s41598-025-20741-z

**Published:** 2025-10-21

**Authors:** Jiaming Qi, Chunyan Song, Hui Liao, Ningxuan Yang, Rui Wang, Jiuming Wang, Boyang Huang, Junjie Guo, Zihan Huang

**Affiliations:** 1 Department of Physics, College of Sciences, Shihezi University, Shihezi, 832000 China; 2 College of Sciences/State Key Laboratory of Advanced Energy Storage Materials and Technologies, Shihezi University, Shihezi, 832000 China

**Keywords:** Al_x_Ga_1−x_N/GaN heterojunctions, First principles, Electronic structure, Thermoelectric properties, Energy science and technology, Materials science, Physics

## Abstract

The effect of low Al concentrations on the electronic structure and thermoelectric properties of Al_x_Ga_1−x_N/GaN (x = 0.1250, 0.1875, 0.2500, and 0.3125) heterojunctions was investigated using density functional theory and Boltzmann transport theory. Compared to Al_x_Ga_1−x_N/GaN heterojunctions with different Al concentrations, it was found that: (1) The bandgap increases and the density of states (DOS) decreases near the Fermi level as the Al concentration increases in Al_x_Ga_1−x_N/GaN heterojunctions. (2) The Seebeck coefficient of the Al_0.25_Ga_0.75_N/GaN heterojunction reaches 1850.20 μV/K at 300 K. (3) For n-type samples, the increase of Al concentration leads to higher conductivity in Al_x_Ga_1−x_N/GaN heterojunctions. (4) Power factor (PF) decreases with increasing Al concentration in Al_x_Ga_1−x_N/GaN heterojunctions. At the lowest Al concentration, the power factor of the Al_0.125_Ga_0.875_N/GaN heterojunction reaches 1.48 × 10^11^W/(m·K^2^·s) at 900K. (5) The maximum electronic thermoelectric quality factor (ZT_e_) of the Al_0.25_Ga_0.75_N/GaN heterojunction reaches 1.41, and at the same temperature, the n-type Al_x_Ga_1−x_N/GaN heterojunctions exhibit significantly higher performance than the p-type. The results are useful for exploring the thermoelectric properties of GaN-based heterojunctions and improving the performance of thermoelectric devices.

## Introduction

Currently, the problem of energy depletion due to the misuse of fossil fuels has become serious, and energy-saving and environmentally friendly thermoelectric materials offer a viable solution to this problem^1–3^. GaN-based semiconductors, with their wide bandgap, high breakdown field, low on-resistance, and high electron mobility^4,5^, have a broad range of applications in optoelectronic devices^6–8^. At the same time, GaN-based semiconductors have good temperature stability and excellent Seebeck coefficients^9,10^. As a result, GaN-based semiconductors have become important candidates for high-temperature thermoelectric applications^11^. However, the conversion efficiency of thermoelectric materials is relatively low compared to conventional energy conversion methods^12^. Therefore, it is essential to improve the thermoelectric properties of these materials.

There are two main methods to enhance the thermoelectric properties of the thermoelectric materials: by reducing the thermal conductivity and increasing the power factor (PF = S^2^σ), currently^13^. Here, S is the Seebeck coefficient and σ is the electronic conductivity. To obtain more efficient thermoelectric materials, heterojunctions composed of basic thermoelectric materials such as GaAs/AlAs^14^, Bi_2_Te_3_/Sb_2_Te_3_^15^, or PbTe/Pb(Sn)SeTe^16^ were investigated. So far, few works have researched the thermoelectric properties of GaN-based heterojunctions. In experimental research, BRYAN et al.^17^ measured the power factor of a two-dimensional n-type AlGaN/GaN heterojunction with an Al concentration of 21.8% to be 3.1 mW/(m·K^2^·s). Kang et al.^18^ characterized and measured the Seebeck coefficient of a 35% high In component two-dimensional InGaN/GaN superlattice to -571V/K.

Density functional theory (DFT)^19^ and Boltzmann transport theory^20^ have been widely applied in theoretical calculations of GaN-based semiconductors. Altaf et al.^21^ calculated the power factor of bulk GaN as 3.0 × 10^10^ μW·cm^−1^·K^−2^·s^-1^. Ji et al.^22^ and Liao et al.^23^ obtained high PF values of bulk GaN and GaN nanowires through the introduction of C-related point defects, respectively. Kaf et al.^24^ calculated the p-type power factor of Al_0.375_Ga_0.625_N using Boltzmann transport theory, which is 64.04 × 10^10^ μW·cm^−1^·K^−2^·s^−1^ at 600 K. Sztein et al.^25^ calculated the Seebeck coefficient of Al_0.3_Ga_0.7_N at 1200 K to be -200 μV/K. In recent years, GaN-based heterojunctions such as g-GaN/g-AlN, AlGaN/GaN et al^26–31^ have attracted much attention in two-dimensional van der Waals heterostructures, UV photodetectors, High Electron Mobility Transistors (HEMTs), and so on. Cong et al.^32^ found the Seebeck coefficient of g-GaN/g-AlN is higher than that of g-AlN and g-GaN. However, the effect of Al concentration on the thermoelectric properties of AlGaN/GaN heterojunctions remains unclear. Therefore, it is necessary to study the effect of Al concentration on the thermoelectric properties of Al_x_Ga_1-x_N/GaN heterojunctions.

In this work, we investigated the effect of low aluminum (Al) concentrations in Al_x_Ga_1-x_N/GaN heterojunctions on the electronic structure and thermoelectric transport properties. The electronic structures including energy band structure and electronic density of states were calculated by density functional theory. Meanwhile, we calculated the thermoelectric transport properties including the Seebeck coefficients, electronic conductivity, and power factor (PF) of Al_x_Ga_1-x_N/GaN heterojunctions, using the Boltzmann transport theory. The effect of different low Al concentrations on the PF of Al_x_Ga_1-x_N/GaN heterojunctions is highlighted.

## Calculation method

**Fig. 1:**
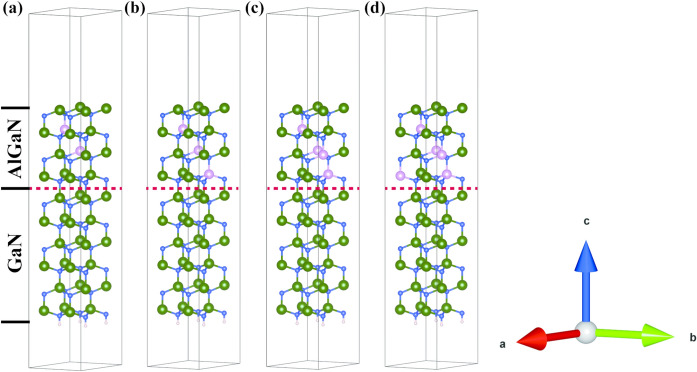
Inclined side view of Al_x_Ga_1-x_N/GaN heterojunctions with (**a**) x = 0.1250, (**b**) x = 0.1875, (**c**) x = 0.2500 and (**d**) x = 0.3125. Red dashed line: interface between Al_x_Ga_1-x_N and GaN, N atoms (blue), Ga atoms (green), Al atoms (pink), and H atoms (white).

The established Al_x_Ga_1-x_N/GaN (x = 0.1250, 0.1875, 0.2500, and 0.3125) structures are shown in Fig. 1 with the interface between Al_x_Ga_1-x_N and GaN indicated by red dashed lines. The structures were modeled based on hexagonal wurtzite GaN primitive cells, with lattice constants of a = b = 3.189 Å, c = 5.186 Å, α = β = 90°, γ = 120°, and space group P6_3_mc^33^. Taking Al_0.125_Ga_0.875_N/GaN for example, the upper part of Fig. 1a shows Al_x_Ga_1-x_N with x = 0.1250, which first expands the GaN primitive cells by 2 × 2 × 2 to obtain GaN supercell, and then randomly replaces 2 Ga atoms with 2 Al atoms to obtain Al_0.125_Ga_0.875_N. In order to eliminate the influence of interface states, the GaN surface AlGaN/GaN heterojunctionsmodel and the AlGaN/GaN heterojunctions described in this work are consistent with the reference1^30^.The lower section of Fig. 1a shows the surface of GaN (0001) with a thickness of six-layers GaN in order to eliminate interlayer coupling^30^. It first expands the GaN primitive cells by 2 × 2 × 3 to obtain a GaN supercell, the cleavs the GaN(0001) surface model, and finally a 15 Å vacuum layer is added to the z-axis and passivated with H passivation at the bottom. Based on the established surface models of Al_0.125_Ga_0.875_N and GaN (0001) surface model, the Al_0.125_Ga_0.875_N/GaN as shown in Fig. 1a was established. Figure 1b–d show that by increasing the concentration of Al on the basis of Al_0.125_Ga_0.875_N, a Al_x_Ga_1-x_N/GaN heterojunction is formed with the GaN (0001) surface model. The total thickness of the Al_x_Ga_1-x_N/GaN heterostructure is 27.89 Å, including 10.36 Å for the Al_x_Ga_1-x_N layer, 15.55 Å for the GaN layer, and 1.98 Å for the interlayer spacing between them.

In this work, all electronic structure calculations were performed in the Vienna ab inito simulation package(VASP)^19,34,35^. We adopted the generalized gradient calculation approximation (GGA) with PBE^35–37^. The Monkhorst-Park special k-points grid was set to 7 × 7 × 1 and numerical integration is awarded in the first Brillouin zone. Al(3s^2^3p^1^), Ga(4s^2^4p^1^), and N(2s^2^2p^3^) are set as valence electrons. To ensure accuracy, the cutoff energy was set to 400 eV and the electron self-consistent convergence criterion was set to 10^–5^ eV. Based on the first-principles calculation results, the thermoelectric transport properties of Al_x_Ga_1-x_N/GaN heterojunctions are calculated by the Boltzmann transport theory.

## Results and discussion

### Electronic band structure

**Fig. 2:**
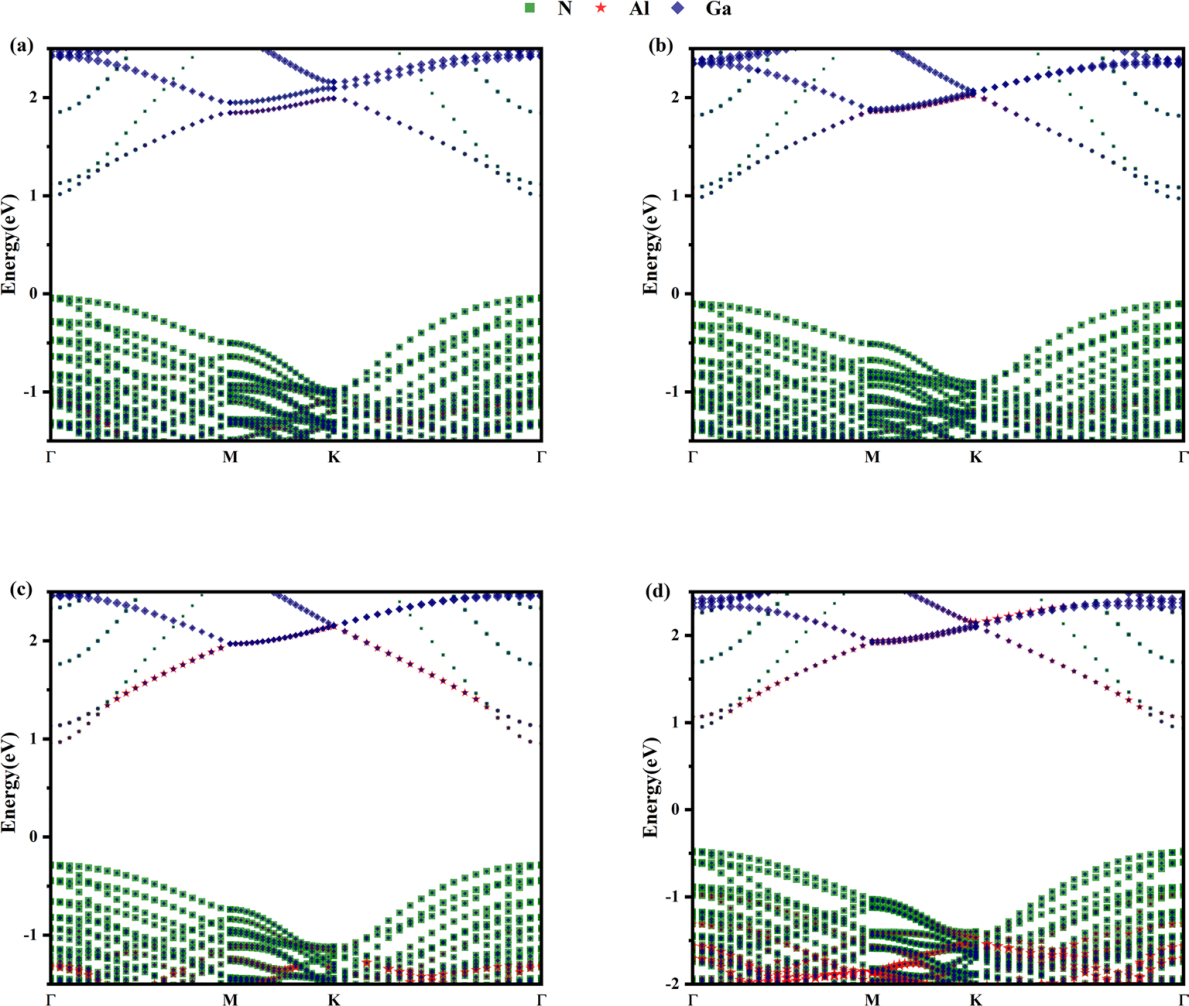
The projection band structures of Al_x_Ga_1-x_N/GaN heterojunctions with (**a**) x = 0.1250, (**b**) x = 0.1875, (**c**) x = 0.2500 and (**d**) x = 0.3125.

We studied the electronic structure of Al_x_Ga_1-x_N/GaN heterojunctions. The projected band structures of Al_x_Ga_1-x_N/GaN heterojunctions with different Al composition were calculated, where x = 0.1250, 0.1875, 0.2500, and 0.3125 in Al_x_Ga_1-x_N/GaN corresponding to (Fig. 2a–d), respectively. We set the Fermi energy level to 0 eV. It was found the four structures of Al_x_Ga_1-x_N/GaN heterojunctions are all direct bandgap semiconductors. We note that as Al composition increases, the bandgap also increases gradually. In the order x = 0.1250, 0.1875, 0.2500, and 0.3125, the bandgaps of Al_x_Ga_1-x_N/GaN heterojunctions are 1.04 eV, 1.07eV, 1.24 eV and 1.41 eV respectively. This is because the increase of Al composition leads to more energy band offset^38^. Figure 2a gives the projected band structure of Al_0.125_Ga_0.875_N/GaN heterojunction. It was found that N acts mainly on the valence band maximum (VBM), while Al and Ga act mainly on the conduction band minimum (CBM), attributed to N’s higher electronegativity. Figure 2b–d give the projected band structures of Al_x_Ga1_-x_N/GaN heterojunctions with x = 0.1875, 0.2500 and 0.3125. With increasing Al concentration, the contribution of Al to CBM increases significantly, while the change in CBM energy decreases slightly with increasing Al concentration. The positions of the CBM relative to the Fermi energy levels are 1.00 eV, 0.97 eV, 0.95 eV, and 0.94 eV, corresponding to x = 0.1250, 0.1875, 0.2500, and 0.3125. Meanwhile, the position of VBM decreases which is -0.04 eV, -0.10 eV, -0.29 eV, and -0.47 eV for the four structures, respectively. This suggests that the Fermi energy level is elevated resulting in a decrease in the VBM with increasing Al concentration. Therefore, the Al_x_Ga_1-x_N/GaN heterojunctions with increasing Al concentration exhibit more pronounced n-type semiconductor characteristics^39^.

### Electron density of states

**Fig. 3:**
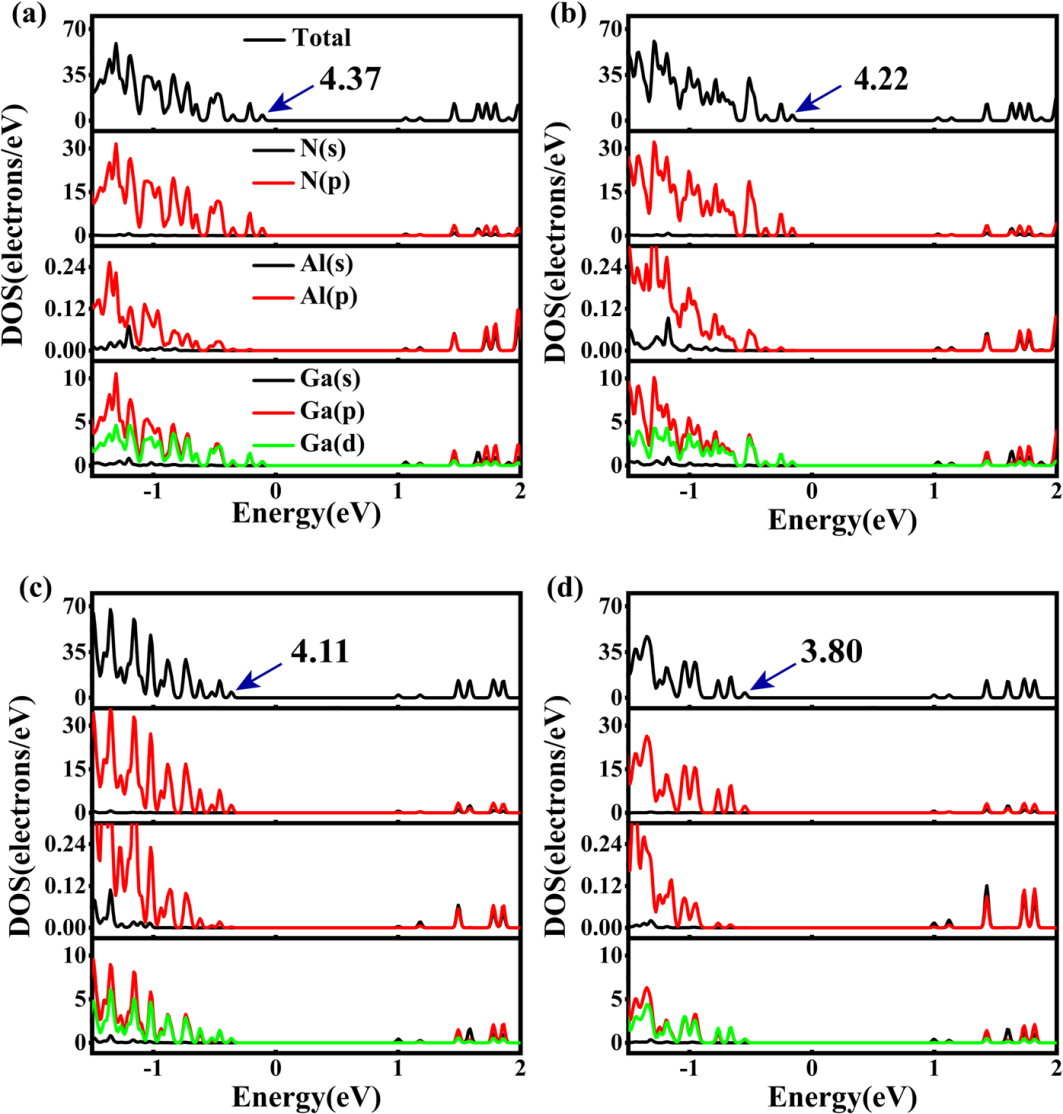
The total density of states (DOS) and the partial electron density of states (PDOS) of Al_x_Ga_1-x_N/GaN heterojunctions with (**a**) x = 0.1250, (**b**) x = 0.1875, (**c**) x = 0.2500 and (**d**) x = 0.3125.

We further investigated the electronic structure of Al_x_Ga_1-x_N/GaN heterojunctions. The density of states (DOS) and the partial electron density of states (PDOS) of Al_x_Ga_1-x_N/GaN heterojunctions with different Al compositions were calculated, where x = 0.1250, 0.1875, 0.2500, and 0.3125 in Al_x_Ga_1-x_N/GaN corresponding to (Fig. 3a–d), respectively. We mainly analyzed the DOS and PDOS near the CBM and the VBM. It was found that all the structures have Ga(p) and Ga(s) contributing predominantly in the CBM and N(p) contributing predominantly in the VBM. Al(p) and Al(s) mainly contribute to CBM, but significantly less than Ga(p) and Ga(s). Meanwhile, the contribution of Al(p) at both the VBM and the CBM rises significantly with increasing Al composition. Concurrently, the Fermi level gradually moves away from VBM, leading to decreased DOS near the Fermi level with increasing Al composition. Near the CBM, the contributions of Al(p) and Al(s) increase, while those of Ga(p) and Ga(s) decrease, and the contributions of N(s) and N(p) remain unchanged, so the overall change in CBM is not significant. This is agrees with the energy band calculations. indicating that the Al concentration has a greater impact on the VBM of Al_x_Ga_1-x_N/GaN heterojunctions.

### Thermoelectric properties

**Fig. 4:**
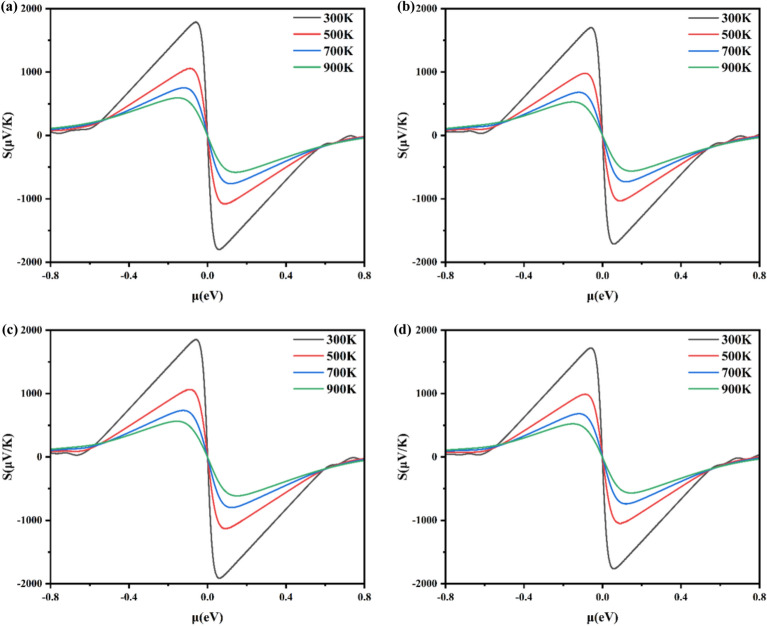
The calculated Seebeck coefficient for Al_x_Ga_1-x_N/GaN heterojunctions with (**a**) x = 0.1250, (**b**) x = 0.1875, (**c**) x = 0.2500 and (**d**) x = 0.3125.

We investigated the thermoelectric properties of Al_x_Ga_1-x_N/GaN heterojunctions, the Seebeck coefficient, electrical conductivity, and the power factor have been computed using Boltzmann transport theory. The Seebeck coefficient and conductivity are important physical quantities for the study of thermoelectric properties. The formula calculated are as follows^40,41^

1 $$S=\frac{8{\pi }^{2}{k}_{B}^{2}}{2e{h}^{2}}{m}^{*}T{\left(\frac{\pi }{3n}\right)}^\frac{2}{3}$$

2 $$\sigma =ne\mu$$

where S is the Seebeck coefficient, k_B_ is the Boltzmann constant, m^*^ is the effective mass, T is the absolute temperature, h is the Planck constant, n is the carrier concentration and μ is the electron mobility. Figure 4 gives the Seebeck coefficient as a function of chemical potential (μ) for different Al concentrations, at 300 K, 500 K, 700 K, and 900 K. For x = 0.1250, 0.1875, 0.2500, and 0.3125 of Al_x_Ga_1-x_N/GaN heterojunctions corresponding to Figs. 4a–d, respectively. Positive chemical potential (μ > 0) corresponds to n-type carriers (electrons), while μ < 0 corresponds to p-type carriers (holes)^42^. As shown in Fig. 4, firstly, the approximate symmetry of the Seebeck coefficients for p-type and n-type of Al_x_Ga_1-x_N/GaN heterojunctions is found. Secondly, the Seebeck coefficient of the Al_0.25_Ga_0.75_N/GaN heterojunction reached 1850.20 μV/K at 300K, compared to 1785.91 μV/K for x = 0.1250, 1699.20 μV/K for x = 0.1875, and 1718.56 μV/K for x = 0.3125 of p-type. For n-type, the Seebeck coefficient of the Al_x_Ga_1-x_N/GaN heterojunctions reaches -1798.92 μV/K, -1711.10 μV/K, -1810.82 μV/K, and -1763.92 μV/K for at 300 K with x = 0.1250, 0.1875, 0.2500 and 0.3125. It can be seen that the Seebeck coefficient remains essentially stable as the Al concentration in the Al_x_Ga_1-x_N/GaN heterojunctions is varied. Thirdly, the Seebeck coefficient decreases with increasing temperature. The maximum value of the p-type Seebeck coefficient for Al_x_Ga_1-x_N/GaN heterojunctions with x = 0.1250 decreases to 523 μV/K at 900 K. This is because the increase in temperature leads to an increase in carrier concentration, and the Seebeck coefficient is inversely proportional to the carrier concentration, resulting in a decrease in the Seebeck coefficient.

**Fig. 5:**
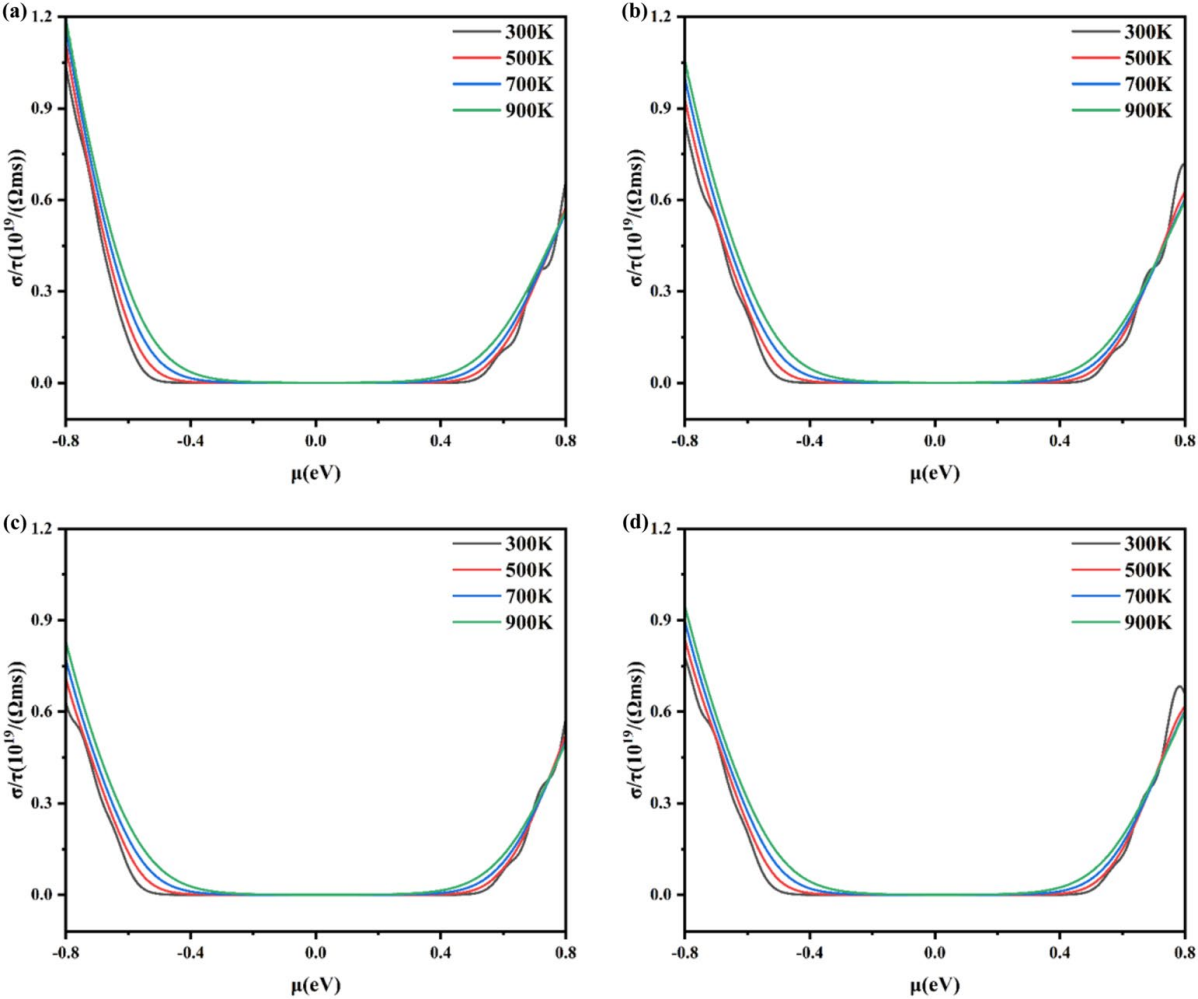
The calculated electrical conductivity for Al_x_Ga_1-x_N/GaN heterojunctions with (**a**) x = 0.1250, (**b**) x = 0.1875, (**c**) x = 0.2500 and (**d**) x = 0.3125.

Figure 5 gives the electrical conductivity as a function of chemical potential (μ) at 300 K, 500 K, 700 K, and 900 K. For x = 0.1250, 0.1875, 0.2500, and 0.3125 of Al_x_Ga_1-x_N/GaN heterojunctions corresponding to (Fig. 5a–d), respectively. Firstly, it was found that temperature changes have little effect on the conductivity. As shown in formula (2), the carrier concentration increases with temperature, but lattice scattering becomes the main mechanism at high temperatures. As the temperature increases, the amplitude of lattice vibration increases, which enhances the scattering effect on carriers and leads to a decrease in mobility. Secondly, Fig. 5 shows that the highest conductivity of p-type Al_x_Ga_1-x_N/GaN is 1.19 × 10^19^/(Ω·m·s) at a temperature of 900 K and x = 0.1250, while it is 1.05 × 10^19^ /(Ω·m·s) for x = 0.1875, 8.2 × 10^18^ /(Ω·m·s) for x = 0.2500, 9.4 × 10^18^/(Ω·m·s) for x = 0.3125 at μ = -0.8 eV. For n-type part, Al_0.3125_Ga_0.6875_N/GaN has the highest electrical conductivity with 6.0 × 10^18^ /(Ω·m·s) compared with 5.6 × 10^18^ /(Ω·m·s) for x = 0.1250, 5.9 × 10^18^ /(Ω·m·s) for x = 0.1875, 5.1 × 10^18^ /(Ω·m·s) for x = 0.2500 at μ = 0.8 eV. Thirdly, the conductivity of the p-type is higher than that of the n-type of Al_x_Ga_1-x_N/GaN, indicating a higher concentration of holes. However, with increasing Al composition, more electrons accumulate at the interface of Al_x_Ga_1-x_N/GaN heterojunctions^39^, leading to a decrease in the difference between the conductivity of p-type and n-type. This is consistent with the result above in Fig. 2 that Al_x_Ga_1-x_N/GaN heterojunctions favor n-type semiconductors.

**Fig. 6:**
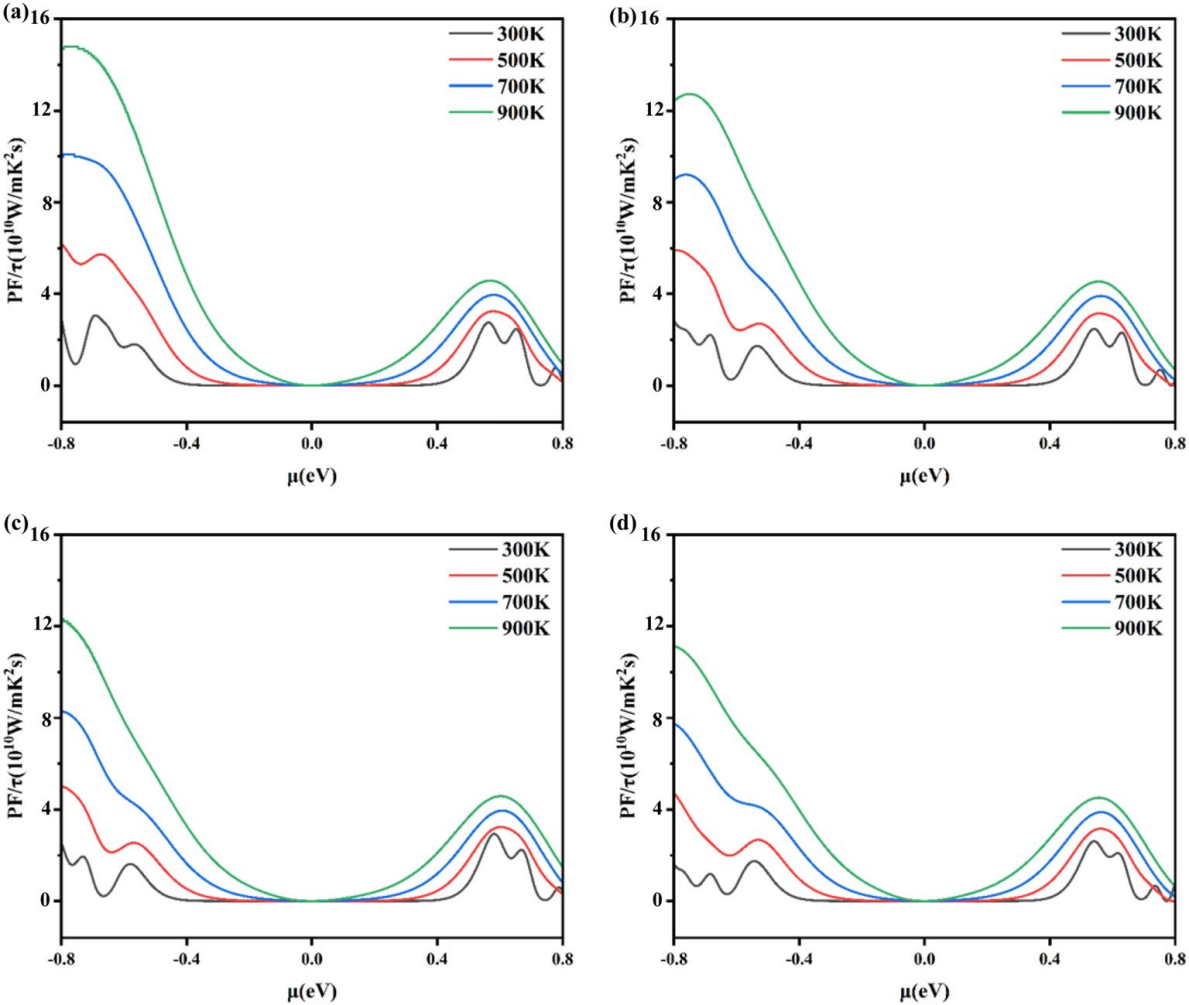
The calculated power factors (PF) for Al_x_Ga_1-x_N/GaN heterojunctions with (**a**) x = 0.1250, (**b**) x = 0.1875, (**c**) x = 0.2500 and (**d**) x = 0.3125.

Figure 6 gives the power factor (PF) as a function of chemical potential (μ) at 300 K, 500 K, 700 K, and 900 K. For x = 0.1250, 0.1875, 0.2500, and 0.3125 of Al_x_Ga_1-x_N/GaN heterojunctions corresponding to (Fig. 6a–d), respectively. Firstly, the p-type PF decreases with the increase of Al composition at the same temperature. Secondly, the PF of all heterojunctions increases with temperature. Thirdly, the maximum values of PF at 300 K are 3.06 × 10^10^W/(m·K^2^·s) for x = 0.1250, 2.79 × 10^10^W/(m·K^2^·s) for x = 0.1875, 2.48 × 10^10^W/(m·K^2^·s) for x = 0.2500, 1.75 × 10^10^W//(m·K^2^·s) for x = 0.3125, respectively. At 900 K, the maximum PF of Al_x_Ga_1-x_N/GaN heterojunctions for x = 0.1250 reaches 1.48 × 10^11^W/(m·K^2^·s), which is higher than in GaN-based semiconductor heterojunctions g-GaN/g-AlN^32^ and ZnO/GaN^43^. This is due to the factor that as Al composition increases in Al_x_Ga_1-x_N/GaN heterojunctions, the Fermi energy level rises shown in Fig. 2, leading to the DOS decreased in Al_x_Ga_1-x_N/GaN heterojunctions at the Fermi energy level^23^, Fourthly, the n-type PF remains stable with Al concentration. At 300 K, the n-type PF for x = 0.1250, 0.1875, 0.2500 and 0.3125 have maximum values of 2.75 × 10^10^W/(m·K^2^·s), 2.57 × 10^10^ W/(m·K^2^·s), 2.78 × 10^10^W/(m·K^2^·s) and 2.61 × 10^10^W/(m·K^2^·s). This also demonstrates that the Al_x_Ga_1-x_N/GaN heterojunctions are good high-temperature thermoelectric materials and that the Al_x_Ga_1__-x_N/GaN heterojunctions with x = 0.1250 has the best thermoelectric properties.

**Fig. 7:**
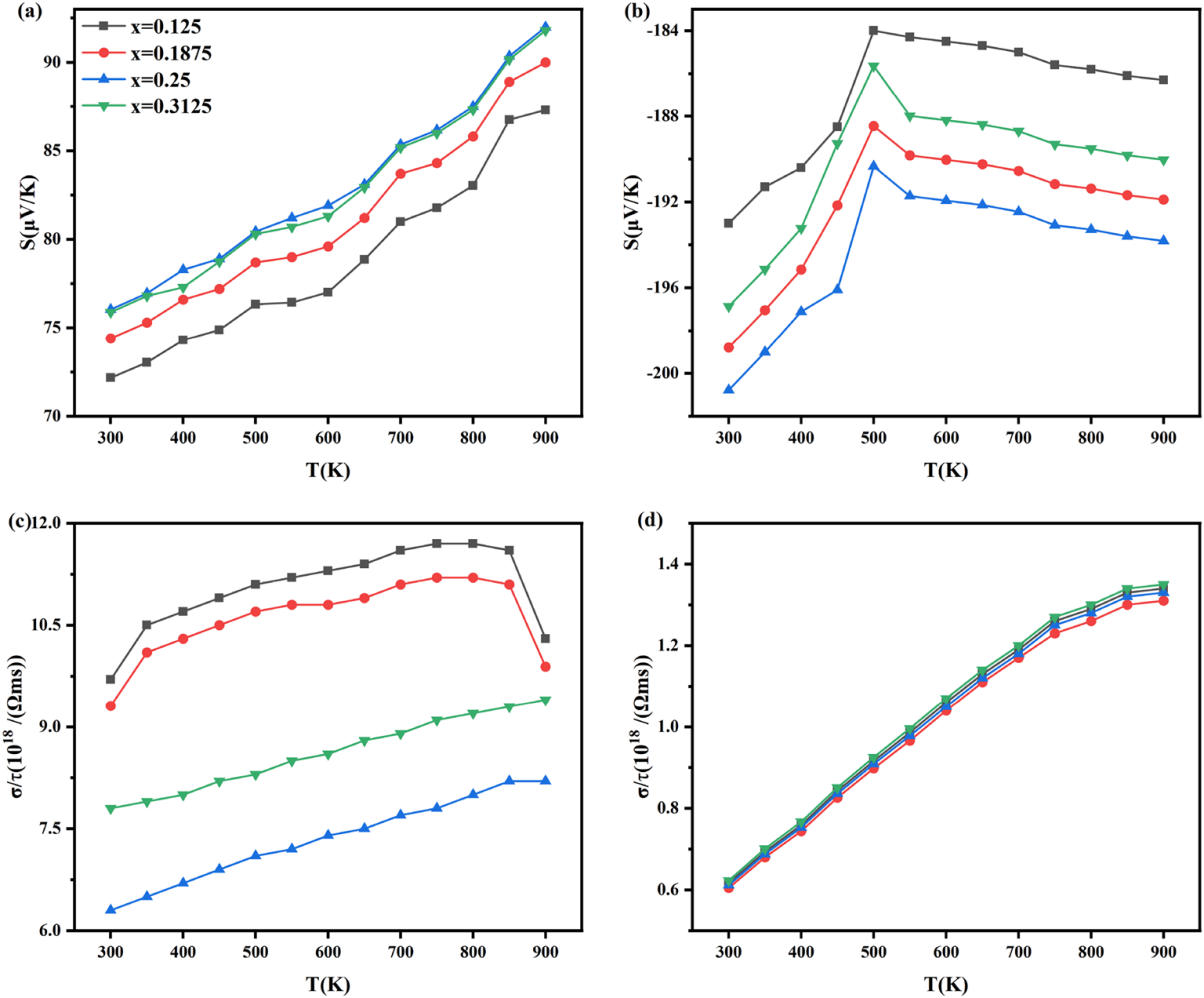
Variation of Seebeck coefficient and conductivity of Al_x_Ga_1-x_N/GaN heterojunctions at PF maxima at different temperatures (**a**) Seebeck coefficients for p-type, (**b**) Seebeck coefficients for n-type, (**c**) conductivity for p-type and (**d**) conductivity for n-type.

In order to better investigate the effects of the Seebeck coefficient and conductivity on the PF of Al_x_Ga_1-x_N/GaN heterojunctions, we counted the Seebeck coefficients and conductivities at the chemical potentials corresponding to the PF maxima at different temperatures, as shown in (Fig. 7). The plots of Seebeck coefficient and conductivity maxima versus temperature for p-type of Al_x_Ga_1-x_N/GaN heterojunctions with different Al concentration are given in (Fig. 7a,c). It was found that the maximum Seebeck coefficient and conductivity do not vary monotonically with Al concentration. As Al concentration increases, the lattice relaxation produces penetrating dislocations. These defects compensate carrier deficiency, increasing carrier concentration. However, serious lattice distortion reduces carrier mobility. At the same time, it can be seen from Fig. 2 that with the increase of Al concentration, the band gap of Al_x_Ga_1-x_N/GaN increases and the curvature decreases, resulting in the increase of effective mass. According to formulas (1) and (2), the Seebeck coefficient is affected by the effective mass and carrier concentration, and the conductivity is affected by the carrier concentration and mobility. Thus, the maximum values of Seebeck coefficient and conductivity do not monotonically change with the concentration of Al. Figure 7 shows that the Al_0.125_Ga_0.875_N/GaN heterojunction has maximum conductivity and thus maximum power factor. The plots of Seebeck coefficient and conductivity maxima versus temperature for n-type of Al_x_Ga_1-x_N/GaN heterojunctions are given in (Fig. 7b,d). Due to large changes in the Seebeck coefficient and small changes in the conductivity occur, while the overall PF change in n-type heterojunctions is insignificant. Therefore, the conductivity dominates PF variation in Al_x_Ga_1-x_N/GaN heterojunctions^44^.

The dimensionless electronic thermoelectric figure of merit (ZT_e_) characterizes the thermoelectric properties of materials, which can be calculated using the following formula^45^:ZT_e_ = S^2^σT/κ_e_. Where S is the Seebeck coefficient, σ is the electrical conductivity, κ_e_ is the electronic thermal conductivity, and T is the absolute temperature.

**Fig. 8:**
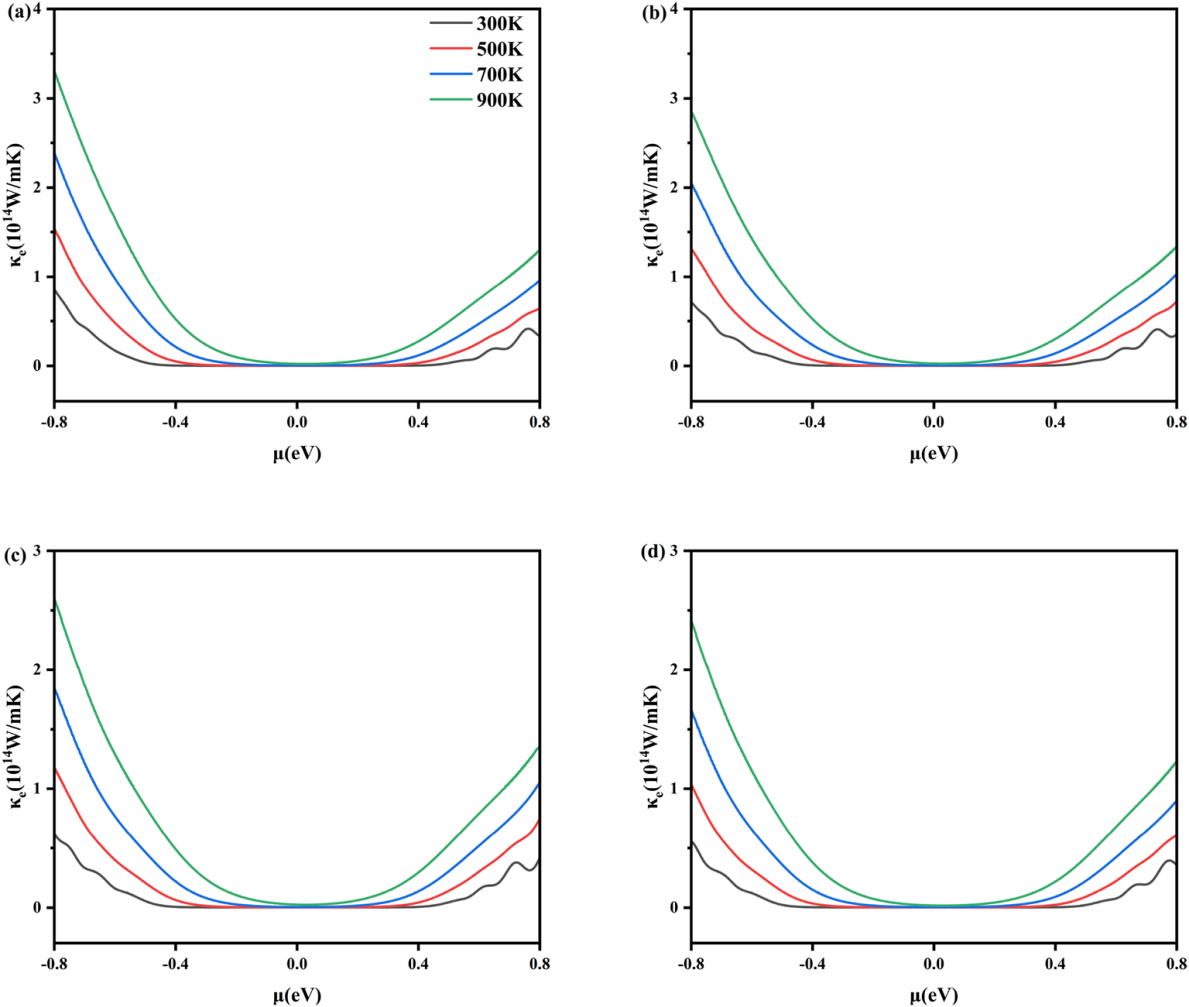
The calculated electronic thermal conductivity for Al_x_Ga_1-x_N/GaN heterojunctions with (**a**) x = 0.1250, (**b**) x = 0.1875, (**c**) x = 0.2500 and (**d**) x = 0.3125.

Figure 8 gives the electronic thermal conductivity (κ_e_) at 300 K, 500 K, 700 K, and 900 K as a function of the chemical potentials (μ) for x = 0.1250, 0.1875, 0.2500, and 0.3125 of Al_x_Ga_1-x_N/GaN heterojunctions corresponding to (Fig. 8a–d), respectively. Firstly, it was found that as the temperature increases, the electronic thermal conductivity of Al_x_Ga_1-x_N/GaN significantly increases. This is because the increase in temperature causes valence band electrons to gain sufficient energy to transition to the conduction band, producing more free electrons and holes, which increases carrier concentration. At the same time, the increase in temperature intensifies the thermal motion of electrons, increases the average kinetic energy, and transfers more thermal energy per unit time. Secondly, Fig. 8 shows that at the same temperature, as the Al concentration increases, the thermal conductivity of p-type and n-type Al_x_Ga_1-x_N/GaN heterojunctions decreases, likely due to stronger phonon scattering caused by higher Al concentration, thereby reducing κ_e_. At 300 K, the electronic thermal conductivity of n-type Al_0.3125_Ga_0.6875_N/GaN decreases to 3.76 × 10^13^ W/m·K. Therefore, increasing the concentration of Al can effectively reduce the electronic thermal conductivity of Al_x_Ga_1-x_N/GaN heterojunctions.

**Fig. 9:**
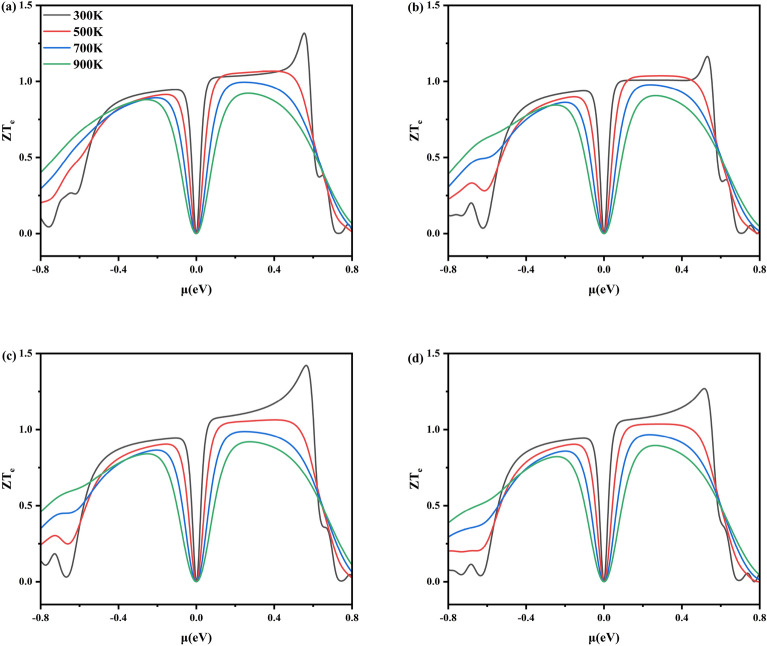
The ZT_e_ for AlxGa1-xN/GaN heterojunctions with (**a**) x = 0.1250, (**b**) x = 0.1875, (**c**) x = 0.2500 and (**d**) x = 0.3125.

Figure 9 gives the electronic thermoelectric figure of merit (ZT_e_) at 300 K, 500 K, 700 K, and 900 K as a function of chemical potential (μ) for x = 0.1250, 0.1875, 0.2500, and 0.3125 of Al_x_Ga_1-x_N/GaN heterojunctions corresponding to (Fig. 9a–d), respectively. Firstly, Because electronic thermal conductivity increases significantly with temperature, ZT_e_ decreases. Secondly, for x = 0.1250, 0.1875, 0.2500, and 0.3125, ZT_e_ reaches 1.32, 1.16, 1.42, and 1.27, respectively, which are all higher than 0.99 for Al_x_Ga_1-x_N materials^24^. Thirdly, at the same temperature, the ZT_e_ of n-type Al_x_Ga_1-x_N/GaN is significantly higher than that of p-type. This result indicates that the Al_x_Ga_1-x_N/GaN heterojunctions exhibits excellent n-type semiconductor performance.

## Conclusion

The electronic structure and thermoelectric transport properties of Al_x_Ga_1-x_N/GaN heterojunctions with low aluminum concentration x = 0.1250, 0.1875, 0.2500, and 0.3125 were investigated by density functional theory and Boltzmann transport theory. Electronic structure show that the Fermi level shifts upward with increasing Al concentration, indicating n-type semiconductor characteristics. Additionally, Al’s contribution to the energy band structure and density of states increases with Al concentration. Due to reduced density of states near the Fermi level, the p-type power factor decreases significantly with higher Al concentrations. Notably, the power factor of Al_0.125_Ga_0.875_N/GaN heterojunction are 1.48 × 10^11^ W/(m·K^2^·s) which is the largest of four samples, so the Al_0.125_Ga_0.875_N/GaN heterojunctions have the best thermoelectric properties. This is the result of the joint regulation of the Seebeck coefficient and the conductivity, where the conductivity plays a major role. In addition, the power factor of Al_x_Ga_1-x_N/GaN heterojunctions increases with increasing temperature, which confirms the potential of Al_x_Ga_1-x_N/GaN heterojunctions as high-temperature thermoelectric materials. At the same time, it was also found at the same temperature, the ZT_e_ of n-type heterojunction is significantly higher than that of p-type heterojunction.

This work will help to improve the thermoelectric performance of GaN-based heterojunctions. In the following research work, the influence of thermal conductivity on thermoelectric properties in GaN-based materials will be further comprehensively considered.

## Data Availability

The datasets generated and analysed during the current study are not publicly available due to their planned use in ongoing computational research by the authors, but are available from the corresponding author on reasonable request.
